# Sex Differences Affect the NRF2 Signaling Pathway in the Early Phase of Liver Steatosis: A High-Fat-Diet-Fed Rat Model Supplemented with Liquid Fructose

**DOI:** 10.3390/cells13151247

**Published:** 2024-07-24

**Authors:** Benedetta Di Veroli, Roger Bentanachs, Núria Roglans, Marta Alegret, Letizia Giona, Elisabetta Profumo, Alessandra Berry, Luciano Saso, Juan Carlos Laguna, Brigitta Buttari

**Affiliations:** 1 Department of Cardiovascular and Endocrine-Metabolic Diseases and Aging, Istituto Superiore di Sanità, 00161 Rome, Italy; benedetta.diveroli@gmail.com (B.D.V.); elisabetta.profumo@iss.it (E.P.); 2 Department of Pharmacology, Toxicology and Therapeutic Chemistry, School of Pharmacy and Food Science, University of Barcelona, 08028 Barcelona, Spain; bentanachs@ub.edu (R.B.); roglans@ub.edu (N.R.); jclagunae@ub.edu (J.C.L.); 3 Institute of Biomedicine, University of Barcelona, 08028 Barcelona, Spain; 4 Spanish Biomedical Research Centre in Physiopathology of Obesity and Nutrition (CIBEROBN), Instituto de Salud Carlos III (ISCIII), 28029 Madrid, Spain; 5 Center for Behavioural Sciences and Mental Health, Istituto Superiore di Sanità, 00161 Rome, Italy; letizia.giona@iss.it (L.G.); alessandra.berry@iss.it (A.B.); 6 Department of Physiology and Pharmacology “Vittorio Erspamer”, Sapienza University, 00185 Rome, Italy; luciano.saso@uniroma1.it

**Keywords:** liver steatosis, fructose, high-fat diet, NRF2, KEAP1, antioxidants, autophagy, endoplasmic reticulum stress response

## Abstract

Sex differences may play a role in the etiopathogenesis and severity of metabolic dysfunction-associated steatotic liver disease (MASLD), a disorder characterized by excessive fat accumulation associated with increased inflammation and oxidative stress. We previously observed the development of steatosis specifically in female rats fed a high-fat diet enriched with liquid fructose (HFHFr) for 12 weeks. The aim of this study was to better characterize the observed sex differences by focusing on the antioxidant and cytoprotective pathways related to the KEAP1/NRF2 axis. The KEAP1/NRF2 signaling pathway, autophagy process (LC3B and LAMP2), and endoplasmic reticulum stress response (XBP1) were analyzed in liver homogenates in male and female rats that were fed a 12-week HFHFr diet. In females, the HFHFr diet resulted in the initial activation of the KEAP1/NRF2 pathway, which was not followed by the modulation of downstream molecular targets; this was possibly due to the increase in KEAP1 levels preventing the nuclear translocation of NRF2 despite its cytosolic increase. Interestingly, while in both sexes the HFHFr diet resulted in an increase in the levels of LC3BII/LC3BI, a marker of autophagosome formation, only males showed a significant upregulation of LAMP2 and XBP1s; this did not occur in females, suggesting impaired autophagic flux in this sex. Overall, our results suggest that males are characterized by a greater ability to cope with an HFHFr metabolic stimulus mainly through an autophagic-mediated proteostatic process while in females, this is impaired. This might depend at least in part upon the fine modulation of the cytoprotective and antioxidant KEAP1/NRF2 pathway resulting in sex differences in the occurrence and severity of MASLD. These results should be considered to design effective therapeutics for MASLD.

## 1. Introduction

Metabolic dysfunction-associated steatotic liver disease (MASLD) has been recently proposed to describe liver disease associated with known metabolic dysfunction [1,2,3].

Many mechanistic and clinical studies support the “multiple parallel hit” hypothesis of MASLD, explaining the interplay among the liver, adipose tissue, and gut in driving inflammation and fibrosis [4]. Physiopathological evidence suggests that energy reserves, glucose, and other carbohydrates such as fructose present in excess can be transformed into lipids that can be stored as triglyceride [5,6]. As a result of metabolic dysregulation, cells cannot cope with the increased lipid flux, leading to lipotoxicity and an overworked β-oxidation pathway. Consequently, chronic lipid localization and reactive oxygen species (ROS) overproduction can perturb fatty acid oxidation, the endoplasmic reticulum (ER) stress response, and mitochondrial function and activate inflammatory cascades, resulting in hepatocyte necroinflammation and collagen deposition, driving further disease progression [7]. Moreover, oxidative stress can aggravate mitochondrial dysfunction, creating a vicious cycle that results in greater lipid buildup and ER stress. [8]. Nuclear factor (erythroid-derived 2)-like 2 (NRF2) plays a vital role in the antioxidant and anti-inflammatory mechanism, activating the antioxidant response elements (AREs), all of which are related to the Kelch-like ECH-associated protein (KEAP1)-NRF2 pathway, which protects hepatic cells from oxidative damage during the development of common chronic liver diseases [9]. Under a steady state, KEAP1 targets NRF2 for ubiquitin/proteasome degradation. In contrast, under conditions of oxidative stress, the activation of NRF2 prompts its translocation into the nucleus, initiating the transcriptional regulation of downstream antioxidant-related factors such as Heme Oxygenase-1 (HO-1) and NAD(P)H:quinone acceptor oxidoreductase 1 (NQO1), thereby renewing the redox balance of cells.

Beyond its important role in the antioxidant system, dietary intervention studies emphasize the regulatory function of NRF2 in lipid metabolism [10,11,12], identifying NRF2 as a potential therapeutic target in MASLD management. However, contrasting results have been reported on the protective effects of NRF2 against nutritional models of diabetes [13] and steatosis [14,15]. A pro-diabetic shift in glucose homeostasis is promoted by the prolonged non-canonical activation of NRF2, via the p62-mediated sequestration of KEAP1 [13]. In a nutritional model of liver steatosis, the different expressions of NRF2 should be explained only in part by the difference in treatment length (short-term vs. long-term), fat composition (saturated vs. unsaturated) [16], or diet components (saturated fats vs. simple sugars-) [17]. For instance, when compared to a high-fat (HF) diet, a long-term high-fructose (HFr) diet led to a more severe steatosis and more pronounced pro-oxidant and proinflammatory signaling activation paralleled by impaired NRF2 nuclear translocation in the HF group.

In response to oxidative damage and proteotoxic stress induced by dietary fructose intake, NRF2 can be activated to coordinate the expression of genes involved in antioxidant defense and in the maintenance of the ER physiology, proteasome and autophagy [18]. The ER stress response, specifically involving the activation of X-box binding protein 1 (XBP1), can promote the expression of NRF2 target genes, providing a coordinated response to ER stress and oxidative stress [19]. Conversely, NRF2 activation has been found to impact the expression of XBP1 and its downstream targets [20]. Moreover, NRF2 is involved in the crucial process of reducing proteotoxic stress by enhancing the expression of pro-survival autophagy. This is achieved through the phosphorylation of the autophagy receptor SQSTM1/p62, particularly in cases where the proteasome pathway’s functionality is compromised [21]. SQSTM1/p62 is a target gene of NRF2, which forms a positive feedback loop [22]. The NRF2 pathway and proteostasis network, influencing each other and cooperating to protect cells from damage, emerge as appealing targets to mitigate the negative effects of fatty liver disease, insulin resistance, and obesity [23,24].

The influence of sex on the development of MASLD is still debated. Human studies show that MASLD exhibits significant sex differences in development and progression, with higher prevalence and severity in men [25]. This disparity is largely attributed to sex hormones, with estrogens playing a protective role by regulating lipid metabolism, suppressing inflammation, and promoting hepatocellular regeneration. Consequently, in pre-menopausal females, the prevalence is low, but it rises after 50 years of age and peaks at the age of 60–69 [26]. We recently reported a striking sex difference in rat metabolism in a nutritional model of simple hepatic steatosis dissociated from obesity [27]. Thus, female Sprague Dawley rats administered a plant-based high-fat diet, abundant in palmitic and stearic acids and lacking cholesterol, along with 10% fructose in the drinking water (high-fat–high-fructose, HFHFr), showed an elevation in liver lipid levels that was attributed to increased de novo lipogenesis (DNL), coupled with reduced fatty acid β-oxidation [28]. Unlike female rats, and despite having similar increases in energy consumption and hepatic DNL, HFHFr males presented hypertriglyceridemia, increased adiposity, and enhanced liver fatty acid β-oxidation, but not hepatic steatosis [27].

Although the role of the KEAP1/NRF2/ARE pathway in liver diseases has been extensively investigated [9], crucial information about the sex-specific signaling pathways that operate at baseline and in diseased states initiated by fructose supplementation is lacking.

Prompted by the evidence that the loss of NRF2 increases sensitivity to MASLD and that potential sex-dependent effects are involved in the metabolic disturbances induced by fructose, the aim of this study was to evaluate in more detail the antioxidant and cytoprotective role of the KEAP1/NRF2 axis and the underlying mechanisms in the HFHFr dietary model using stored liver samples from our previous studies in female and male rats [27,28].

## 2. Materials and Methods

### 2.1. Animals and Diets

Male and female Sprague Dawley rats, aged two months, were purchased from Envigo, Barcelona, Spain. They were housed in cages, with two per cage, and randomly assigned to two groups (8 rats for group). The control group (Ctrl) was fed a standard chow diet ad libitum (2018 Teklad Global rodent diet, Envigo, Barcelona, Spain) and had unrestricted access to water. The HFHFr group was given a high-fat diet ad libitum (Teklad Custom Diet TD. 180456, Envigo, Madison, WI, USA) and had unrestricted access to a 10% *w*/*v* fructose solution. The initial weight of the animals was 179 ± 9 g (females) and 228 ± 13 g (males), and the diets were administered for three months. The compositions of Ctrl and HFHFr diets were the same as those previously reported [27,28]. All animals were exposed to constant humidity (40–60%) and temperature (20–24 °C), with a 12 h light/12 h dark cycle. Fructose beverages were replaced and monitored three times per week. Solid food intake and body weight were recorded weekly. All procedures followed the guidelines set by the Bioethics Committee of the University of Barcelona (Autonomous Government of Catalonia Act 5/21 July 1995) and were approved by the Animal Experimentation Ethics Committee of the University of Barcelona (approval no. 10106). At the conclusion of the treatment, all rats were fasted for 2 h, and blood, serum, and tissue samples were collected as previously described [28].

### 2.2. RNA Preparation and Analysis

Total RNA was isolated from liver tissue using “Total RNA Purification Kit” (Norgen Biotek, Thorold, ON, Canada), according to the manufacturer’s instructions. The RNA concentration and purity were measured spectrophotometrically using Nanodrop NanoPhotometer^®^ N60/N50 (Implen GmbH, Munich, Germany). The ratios of absorbance at 260/230 and 260/280 were used as indicators of RNA purity. Total RNA was retrotranscribed to cDNA using SensiFAST cDNA Synthesis Kit (Meridian Life Science Inc., Memphis, TN, USA) including reverse transcriptase, random primers, and a buffer, according to manufacturer’s instructions. The cDNA was produced through a series of heating and annealing cycles in the thermocycler LineGene 9600 Plus (Bioer Technology, Hangzhou, China). Quantitative real-time PCR (qPCR) was performed for each sample in triplicate and reactions were carried out in a 20 µL reaction volume on 96-well plates on LineGene 9600 Plus Fluorescent Quantitative Detection System (Bioer Technology) using cDNA and SensiFAST^TM^ SYBR^®^ No-ROX Kit (Bioline, London, UK). Real-time PCR was carried out using the following cycling conditions: 1 cycle of polymerase activation at 95 °C for 2 min, 40 cycles of denaturation at 95 °C for 5 s, and annealing and extension at 60 °C for 20 s. The primer sequences for the rat genes tested and the internal reference gene *Gapdh* are shown in Table 1.

A comparative threshold cycle (CT) method was used to analyze the real-time PCR data, where the amount of the target, normalized to the endogenous reference of *Gapdh* (ΔCT) as reported by Martínez-Beamonte et al. [29] and relative to the calibrator of untreated control (ΔΔCT), was calculated using the equation 2^−ΔΔCT^, as previously described in the literature [30], where ΔCt = Ct(target) − Ct(Gapdh).

### 2.3. Nuclear and Cytosolic Proteins Extraction

Minute™ Cytosolic and Nuclear Extraction Kit for Frozen/Fresh Tissues (Invent Biotechnologies Inc., Rochester, MN, USA) was utilized for frozen liver tissues. The protease and the phosphatase inhibitors (1 mM sodium fluoride, 1 mM sodium ortho vanadate, and 1 mM sodium molybdate; 1 mM phenylmethylsulfonyl fluoride and 1 mM phosphoinositidase C; Sigma-Aldrich, Milan, Italy) were added to the protein extraction steps. All the procedures were conducted in accordance with the manufacturer’s instructions. Both cytoplasmic and nuclear extracts were used to determine the total protein concentration via the BCA method (Pierce, Rockford, IL, USA).

### 2.4. Western Blotting Analysis

Protein samples (20 μg) were separated via 4–15% sodium dodecyl sulfate-polyacrylamide gel electrophoresis (SDS-PAGE) using Mini-PROTEAN^®^ TGX Stain-Free™ Precast Gel (Bio-Rad Laboratories, Hercules, CA, USA) and transferred onto PVDF membranes (Millipore, Milan, Italy) using Trans-Blot Turbo Transfer System (Bio-Rad Laboratories).

The blot membrane was imaged by the ChemiDoc MP imaging system (Bio-Rad Laboratories) using the stain-free blot settings for the protein total load, which was used later to normalize the data by eliminating sample loading variability. To prevent non-specific antibody binding, the membrane was incubated for 1 h at room temperature in a solution of 5% (*w*/*v*) low-fat milk (Sigma-Aldrich) and 1% (*w*/*v*) bovine serum albumin (Sigma-Aldrich) in Tris-buffered saline (137 mM NaCl, 20 mM Tris·HCl, pH 7.6) with 0.1% Tween 20 (Sigma-Aldrich) (TBS-T). The membrane was incubated overnight at 4 °C with the following primary antibodies: NRF2 (SC-365949, Santa Cruz Biotechnology, Dallas, TX, USA; 1:500), KEAP1 (PA5-34454, Invitrogen, Waltham, MA, USA; 1:500), HO-1 (Bioss Inc., Woburn, MA, USA; 1:500), NAD(P)H quinone oxidoreductase 1 (NQO1) ([A180], Abcam, Waltham, MA, USA; 1:500), p62/SQSTM1 (2C11, Abnova, Hsinchu 300, Taiwan; 1:500), LC3B (NB100-2220, Novus Biologicals, Centennial, CO, USA; 1:500), XBP1 (A17007, Abclonal, Abclonal Technology, Chongqing, China; 1:500), Calpain (SC-271856 B8, Santa Cruz, 1:500), Histone H1(SC-393358, Santa Cruz, 1:500), and β-actin (Sigma-Aldrich, 1:1000). The proteins of interest were detected using appropriate peroxidase-conjugated secondary antibodies (Bio-Rad, 1:5000 dilution) and enhanced chemiluminescence (Clarity Western ECL Substrate; Bio-Rad). Blots were then imaged by the ChemiDoc MP imaging system using chemiluminescence settings. Subsequent determination of relative abundance via total protein normalization was achieved using Image Lab 6.1 software (Bio-Rad Laboratories).

### 2.5. Statistical Analysis

The statistical analysis was performed by GraphPad Prism 8 software (San Diego, CA, USA). Normally distributed data were analyzed using an exploratory 2-way-ANOVA with the HFHFr diet and sex as between-subjects factors. If ANOVA showed a significant sex × diet interaction and/or a significant sex or diet effect, a Tukey post hoc test or independent t-test was conducted for group comparisons. The Shapiro–Wilk test was applied to test data normality. Correlation analyses were performed and graphically presented using the corr.test() and corrplot() functions in R version 4.2.2 (R Foundation for Statistical Computing, Vienna, Austria). Values of *p* < 0.05 were considered statistically significant.

## 3. Results

### 3.1. KEAP1/NRF2 Expression

To evaluate the role of the KEAP1/NRF2 axis and the sex-specific differences in a rat model of MASLD (the dietary HFHFr model), we used stored liver samples from our previous studies in female and male rats [27,28]. These studies showed that when compared to rats on a control diet, male rats exhibited only a slight increase in liver triacylglycerol levels and no signs of hepatic steatosis, as determined by the Oil Red O (ORO) staining of liver sections [18], whereas in female rats the same diet led to severe steatosis, evidenced by an 11-fold increase in liver ORO staining [28]. The characterization of the model in each sex is extensively described in each publication, and a summary of the basic metabolic data of the studied experimental groups is provided in Supplementary Material Table S1.

**Figure 1:**
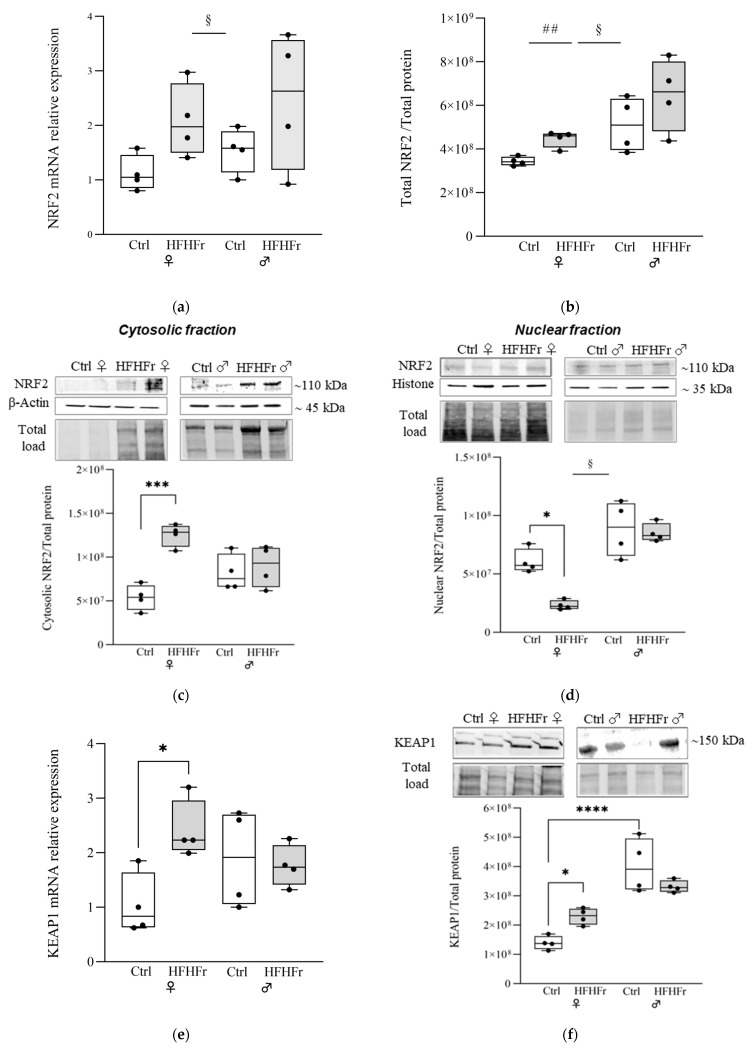
Effect of HFHFr diet on the expression levels of the NRF2 and NRF2 regulator KEAP1 in the liver of female (♀) and male (♂) rats. We used eight animals for each experimental group and prepared four different pooled samples for each experimental group (final N = 4): (□) control (standard rodent chow and water) or (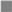) HFHFr (high-fat diet devoid of cholesterol, plus 10% fructose as beverage); each pool was obtained from mixing equal amounts of two individual tissue samples. The box plot graphs show medians and inter-quartile ranges of NRF2 quantification at the gene expression level (**a**), at protein levels as the total NRF2 (**b**), in the cytosolic (**c**) and nuclear fractions, and (**d**) in the liver of female and male animals after being fed the HFHFr diet and control diet. (**c**,**d**) Western blot representative images and densitometric analysis. (**e**,**f**) KEAP1 quantification at the gene expression level and protein level. Comparison among groups was carried out via 2-way ANOVA using the HFHFr diet and sex configuration. A significant main effect of diet is indicated by §; a significant main effect of sex is indicated by #; A Tukey post hoc test or independent *t*-test was conducted for group comparisons and are indicated by *. Significant differences are indicated by *p*-values: *,§ *p* < 0.032; ## *p* < 0.0021; *** *p* < 0.0002; **** *p* < 0.0001.

To decipher the regulatory function of the HFHFr diet in the NRF2 pathway, the mRNA levels of genes known to influence the activation of the KEAP1-NRF2-ARE pathway were measured in the liver of Ctrl and HFHFr groups from female and male rats. Subsequently, the levels of proteins encoded by the mRNA showing differential expression were evaluated using the Western blotting assay. As shown in Figure 1a,b, the HFHFr diet increased both mRNA (main effect of diet: F_(1, 12)_ = 6.227; *p* = 0.0282) and protein levels (main effect of diet: F_(1, 12)_ = 5.040; *p* = 0.0444) of NRF2 in the liver; moreover, males were characterized by higher levels of NRF2 total protein than females (main effect of sex: F_(1, 12)_ = 12.14; *p* = 0.0045; sex x diet interaction: F_(1, 12)_ = 0.1041; *p* = 0.7525).

A more detailed localization of the expression of NRF2 (95–110 kDa) was obtained via Western blot analysis of the abundance of NRF2 in the nuclear and cytosolic fractions extracted from rat livers. HFHFr reduced NRF2 in the nuclear fractions in both females and males (main effect of diet: F_(1, 12)_ = 6.227; *p* = 0.0282). However, in the cytosolic fractions, NRF2 was significantly increased in HFHFr females only (sex x diet interaction: F_(1, 12)_ = 11.66; *p* = 0.0051) (Figure 1c,d).

It is widely accepted that KEAP1 sequesters and represses NRF2 in the cytoplasm, thus preventing its translocation to the nucleus. Significant sex x diet interactions were found both at the protein (F_(1, 12)_ = 10.21; *p* = 0.0077) and mRNA level (F_(1, 12)_ = 5.757; *p* = 0.0336) of KEAP1. In particular, we found that KEAP1 mRNA and protein expression was significantly increased by HFHFr in females only, whereas there was no significant change in the male group (Figure 1e,f). Of note is that the basal levels of KEAP1 protein were higher in males as compared to those in females (Figure 1f).

### 3.2. Antioxidant Expression Levels

**Figure 2:**
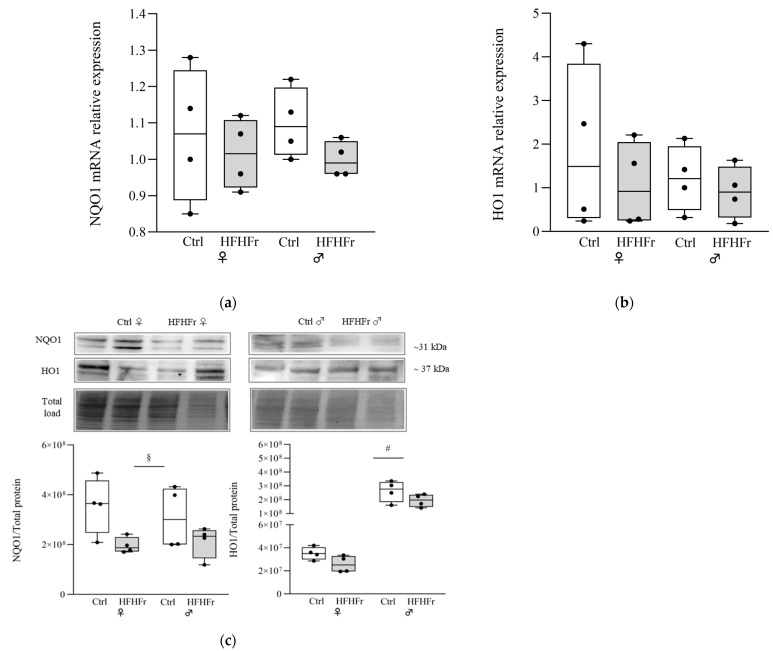
Effect of HFHFr diet on the expression levels of antioxidants in the liver of female (♀) and male (♂) rats. We used eight animals for each experimental group and prepared four different pooled samples for each experimental group (final N = 4): (□) control (standard rodent chow and water) or (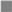) HFHFr (high-fat diet devoid of cholesterol, plus 10% fructose as beverage); each pool was obtained from mixing equal amounts of two individual tissue samples. The box plot graphs show medians and inter-quartile ranges of NQO1 and HO1 quantification at the gene expression level (**a**,**b**), and at protein levels (**c**), in the liver of female and male animals after being fed the HFHFr diet and control diet. (**c**) Western blot representative images and densitometric analysis. A significant main effect of sex is indicated by # and the main effect of diet is indicated by §. Significant differences are indicated by *p*-values: §,# *p* < 0.032.

We next investigated whether the reduced NRF2 nuclear level found in the female HFHFr group was associated with changes in the antioxidant proteins NQO1 and HO-1, the expression of which is regulated by NRF2. A main effect of diet was detected for NQO1 at the protein level (main effect of diet: F_(1, 12)_ = 7.813; *p* = 0.0162; sex x diet interaction: F_(1, 12)_ = 0.4739; *p* = 0.5043), with HFHFr subjects showing reduced expression of NQO1 protein levels. As for HO-1, a significant effect of sex was found (F_(1, 12)_ = 7.759; *p* = 0.0165; sex x diet interaction: F_(1, 12)_ = 3.141; *p* = 0.1017) with males being characterized by a significantly greater expression of HO-1 at the protein level when compared to the female groups (Figure 2c).

### 3.3. Autophagic Pathway

**Figure 3:**
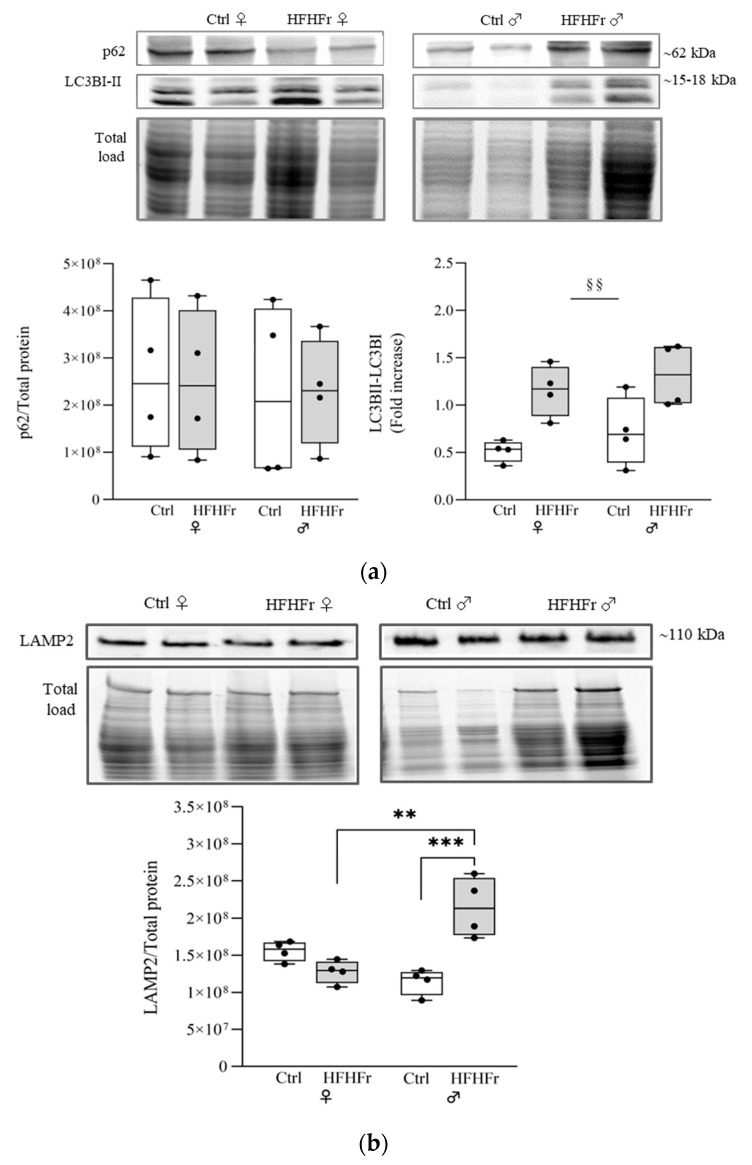
Effect of the HFHFr diet on the expression levels of autophagy markers in the rat livers. The box plot graphs show medians and inter-quartile ranges of p62, LC3B and LAMP2 quantification via Western blotting in the liver of female (♀) and male (♂) rats after being fed a (□) control (standard rodent chow and water) and (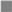) HFHFr (high-fat, devoid of cholesterol, plus 10% fructose as beverage) diet. Western blot representative images and densitometric analysis of (**a**) p62 and LC3B and (**b**) LAMP2 are shown. We used eight animals for each experimental group and prepared four different pooled samples for each experimental condition; each pool was obtained by mixing equal amounts of two individual tissue samples (final N = 4). A significant main effect of diet is indicated by §. Tukey post hoc tests were conducted for group comparisons, and the results are indicated by *. Significant differences are indicated by *p*-values: §§,** *p* < 0.0021; *** *p* < 0.0002.

We next investigated the expression of p62 and LC3B, critical proteins that serve as bridges between the NRF2 pathway and autophagy [31,32], via Western blotting. It has been reported that p62 and NRF2 appear to exert a synergistic effect in protecting hepatocytes from lipotoxicity and the development of liver steatosis [33,34]. The Western blot analysis of p62 did not show a significant difference due to diet or sex (Sex x diet interaction: F_(1, 12)_ = 0.008855; *p* = 0.9266), while a significant increase in the ratio of LC3BII to LC3BI was observed in both sexes in response to the HFHFr diet (main effect of diet: F_(1, 12)_ = 18.60; *p* = 0.0010; sex x diet interaction: F_(1, 12)_ = 0.01948; *p* = 0.8913) (Figure 3a).

To exclude the possibility of the impairment of autophagic flux in late events, such as autophagosome–lysosome fusion, we determined the levels of LAMP2, an important protein for the progression of autophagy and protection against various peroxidation injuries [35]. Western blot analysis revealed a significant increase in LAMP2 in the liver of male rats in response to the HFHFr diet (sex x diet interaction: F_(1, 12)_ = 28.14; *p* = 0.0002) (Figure 3b); no effect of diet was observed in female rats.

### 3.4. Endoplasmic Reticulum Responses

**Figure 4:**
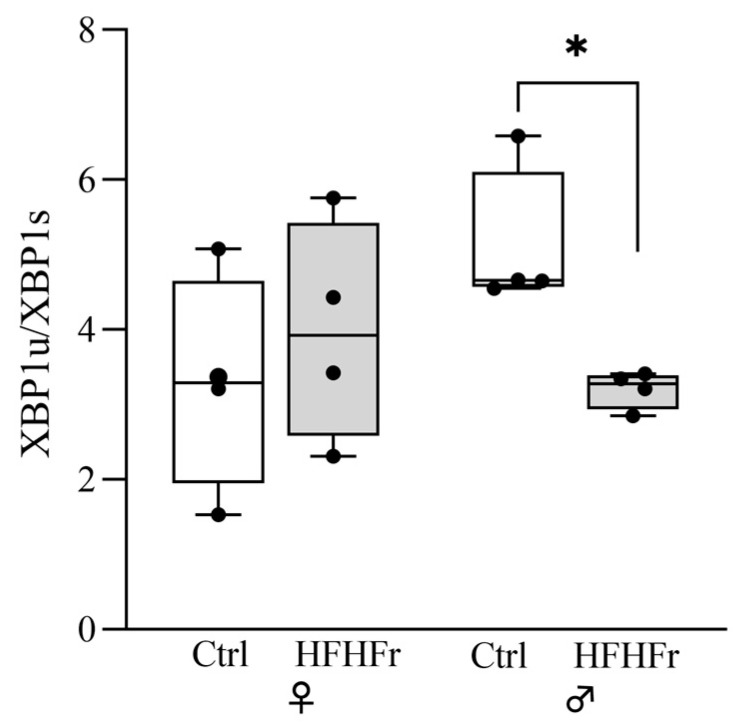
Effect of HFHFr diet on the expression levels of unfolded protein response stress sensor XBP1 in the rat livers. The box plot graphs show medians and inter-quartile ranges of the XBP1u/XBP1 ratio quantification in the liver of female (♀) and male (♂) rats after being fed a (□) control (standard rodent chow and water) and (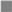) HFHFr (high-fat, devoid of cholesterol, plus 10% fructose as beverage) diet. We used eight animals for each experimental group and prepared four different pooled samples for each experimental condition; each pool was obtained by mixing equal amounts of two individual tissue samples (final N = 4). Significant differences are indicated by *p*-values: * *p* < 0.05.

X-box binding protein 1 (XBP1) is a key molecule governing the oxidative stress and endoplasmic reticulum (ER) stress responses, protecting cells by directly targeting several antioxidant genes [19]. Under stress, XBP1u mRNA (the unspliced form) is induced by ATF6 and successively spliced by IRE1 in a highly active transcription factor termed XBP1s [36]. Figure 4 shows a significant decrease in the XBP1u/XBP1s ratio in male rats only fed the HFHFr diet (sex x diet interaction: F_(1, 12)_ = 5.077; *p* = 0.0437; *t*-tests: *p* < 0.032). This ratio reflects an increase in the spliced XBP1s in the face of XBP1u (Figure S1). Additionally, among the control and HFHFr groups, protein levels of both XBP1 forms in the female group were higher than those in the male group (Figure S1), thus overall suggesting the more active endoplasmic reticulum stress responses of males to the HFHFr diet.

### 3.5. Correlation Analysis

**Figure 5:**
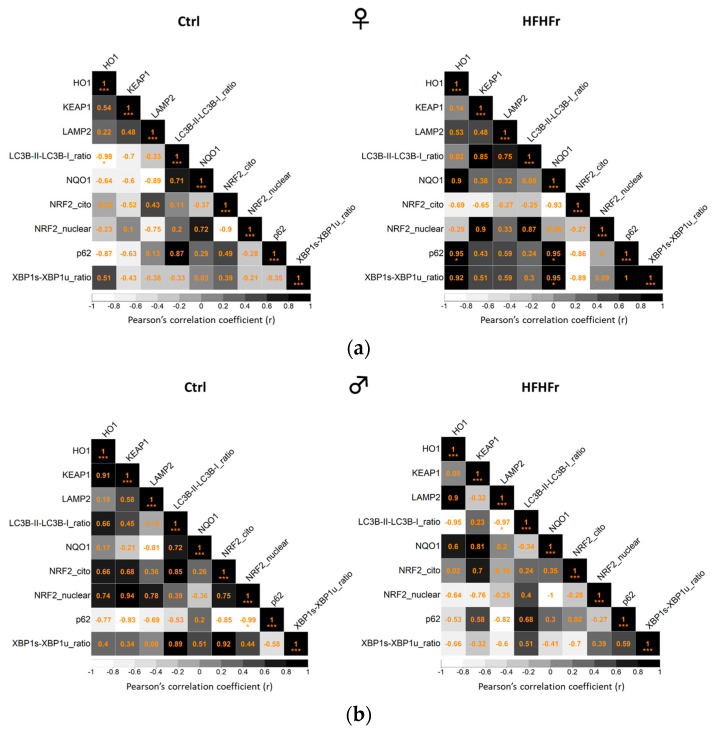
Correlation analysis of the NRF2, KEAP1, NRF2-mediated antioxidant defense response; ERS- and autophagy-related proteins detected via Western blot in the liver of female (♀, **a**) and male (♂, **b**) rats after being fed a control (standard rodent chow and water) and HFHFr (high-fat, devoid of cholesterol, plus 10% fructose as beverage) diet. Significant correlations are indicated by *p*-values: * *p* < 0.05, *** *p* ≤ 0.05. The color gradient reflects correlation coefficients (white = strong negative correlation; black = strong positive correlation).

Pearson’s method (Pearson’s correlation coefficient) was used to further verify the association among NRF2, KEAP1, the NRF2-mediated antioxidant defense response, autophagy, and the ER stress response. As shown in Figure 5, in the female HFHFr group, p62 (autophagy-related protein) and the XBP1u/XBP1s ratio (ER stress response protein) were both positively correlated with both HO-1 and NQO1 (NRF2-mediated antioxidant defense response), thus suggesting the activation of the main cytoprotective signaling pathways to counteract tissue damage. Interestingly, in the male HFHFr group, there was a significative negative correlation between the ratio of LC3BII to LC3BI (autophagosome formation) and LAMP2 (autophagosome–lysosome fusion) protein levels, thus suggesting functional autophagic flux.

## 4. Discussion

In this study, we provided evidence that the KEAP1/NRF2 axis plays a role in the previously observed female-specific vulnerability to MASDL in response to a metabolic insult, such as that induced by the long-term administration of a HFHFr diet [28]. Indeed, in our previous studies, we showed that HFHFr-fed female rats developed steatosis in association with increased lipogenesis, coupled with reduced fatty acid β-oxidation [28]. In contrast, HFHFr males showed a more resilient metabolic phenotype, as they were characterized by lower hepatic lipid accumulation despite similar increases in energy consumption, hepatic DNL, hypertriglyceridemia, increased adiposity, and enhanced liver fatty acid β-oxidation [27]. Here, we show that the female liver phenotype is also characterized by increased levels of cytosolic NRF2 and a reduction in NRF2 nuclear localization, along with an increased expression of KEAP1 (an inhibitor of NRF2). This result is in line with the upregulation of NRF2 expression levels observed in the liver of patients with nonalcoholic fatty liver disease (NAFLD) and diet-induced obesity mouse models, suggesting an involvement of the NRF2/KEAP1 pathway in the regulation of hepatic lipid metabolism [37]. The observed reduction in NRF2 nuclear localization suggests that long-term high-fat and -fructose diet feeding induces NRF2 transcription and its accumulation in the cytosol, in both the phosphorylated and nonphosphorylated forms, but also impairs nuclear translocation of the phosphorylated NRF2, possibly as a result of oxidative damage [38]. The impairment in NRF2 nuclear translocation is also in line with the study by Nigro et al. [17], which found similar results in a mouse model of MASLD.

Our findings on the inhibition of NRF2 nuclear translocation in females are in accordance with the fructose survival hypothesis of obesity suggesting that metabolic disorders and obesity result from the over-stimulation of an evolutionary-based biologic response aimed at protecting animals in advance of crisis [39]. Thus, fructose consumption drives energy imbalance by reducing the active energy (adenosine triphosphate) in the cell and by blocking its regeneration from fat stores.

In this study, all males were characterized by higher NRF2 expression levels in the nuclear fraction than females, possibly representing an increased intrinsic signaling capacity to counteract oxidative stress.

In this sense, compared to females, the expression levels of the antioxidant enzyme HO-1—which is modulated by NRF2 [40]—were higher in males, possibly suggesting a greater ability to face liver injury and fibrosis in this sex and confirming previous results of a male resilient phenotype [27].

Analysis of autophagic pathways in the liver also revealed interesting sex-different strategies in response to an HFHFr insult. Autophagy serves as a crucial cellular protection mechanism, and its suppression in hepatocytes can result in the accumulation of lipids in the liver [41,42]. In the current study, fructose supplementation impaired liver autophagy specifically in female rats. To elaborate on this, while the activation of autophagy is observed in both sexes (as a result of LC3 upregulation), only males show proper authophagic flux (as by the increase in LAMP2 expression), while this is impaired in females. Despite this evidence, here, we failed to confirm the accumulation of p62 in females, as previously observed as a result of a 8-week feeding with normal chow supplemented with liquid fructose [43]. In this regard, we can hypothesize that either the addition of a high-fat diet to the fructose supplement, or the prolonged administration (12 weeks) might have resulted in an imbalance between autophagosomes/autolysosomes in favor of the autophagosomes in females.

Increasing evidence suggests that the ER stress response can trigger autophagy and apoptosis [44]. In line with the above-mentioned results, we found a decrease in the XBP1u/XBP1s ratio upon the administration of the HFHFr diet specifically in males, suggesting a more efficient coping strategy of the hepatocytes in response to a prolonged metabolic insult. In contrast, the lack of changes in the levels of XBP1 observed in females is in line with our previous study involving female rats, which showed a specific increase in phosphorylated IRE-1 (a splicing enzyme for XBP1) levels due to the HFHFr diet, despite the fact that such activation was not followed by a parallel increase in the levels of XBP1 [28]. Further studies will need to provide insights into the regulation of the ER stress response in the HFHFr diet in both sexes in order to provide crucial insights into the underlying mechanisms in the metabolic disorders induced by fructose consumption and potentially identify sex-specific therapeutic strategies. Overall, our results point to reduced protection against pro-oxidant injuries in females, further supporting a sex asymmetry in the intracellular signaling pathways in response to the metabolic disturbances induced by fructose. Correlation analysis showed that in HFHFr females, the levels of the p62 and of XBP1u/XBP1 ratio were both positively related to HO-1 and NQO1 expression. Conversely, a strong negative correlation was observed between the LC3BII/ LC3BI ratio and LAMP2 expression in males. These findings suggest that NRF2-mediated antioxidant defense, autophagy, and ER stress responses were significantly impaired by the HFHFr diet in both sexes and confirm that this impairment should specifically affect females. Thus, it can be speculated that the perturbations in lipid homeostasis induced by the HFHFr diet activate the expression of genes that encode functions to mitigate the effects of excess lipids and limit mitochondrial dysfunction in rat livers. Indeed, energy metabolism is a finely regulated process in mammals possibly as a result of sex-specific and evolutionary conserved mechanisms accounting for gestation and lactation in females while rather reflecting a steady state in males. In general, females are characterized by increased lipids storage and greater insulin sensitivity; by contrast, males show increased visceral adiposity and lipid oxidation, conditions influenced by sex chromosomes and hormones. These considerations might provide a possible explanation for observed different sex-dependent effects in response to the HFHFr metabolic challenge [27,28,45].

A limitation of our work is the reduced number of samples analyzed due to the application of the cost-effective strategy of sample pooling that led to increased heterogeneity in the measured outcomes, thus reducing the ability to detect subtle differences between samples. Further studies using long fructose supplementation associated with a plant-based fat should be performed in other rat strains to ascertain whether these apparent sex differences observed in the development of MASLD situation persist in other genetic backgrounds, although prior research showed that male Sprague Dawley and Wistar rats experienced comparable qualitative metabolic changes when subjected to a high-fat diet [46]. These limitations highlight the need for the careful consideration and interpretation of results obtained from these animal models. Further research is needed to accurately investigate the connection between the long-term consumption of fructose-sweetened beverages and steatosis liver disease in human populations, based on epidemiological evidence.

## 5. Conclusions

To our knowledge, this is the first study providing evidence of a sex dimorphism of the KEAP1/NRF2 pathway in the liver (both at transcriptional and protein levels) in response to HFHFr chronic metabolic challenges. In particular, our result suggest that males are characterized by an improved ability to cope with an HFHFr metabolic stimulus mainly through an autophagic-mediated proteostatic process, while in females, this is impaired. These results should be considered to design effective therapeutics for MASLD. Although the results of our work cannot be directly generalized to humans, they demonstrate the need to explore the sex differences underlying the underestimated effect of sex dimorphism in fructose metabolism and NRF2 responsiveness. It is essential to understand and define sex-specific signaling pathways that function under normal conditions and in diseased states. Sex differences should be taken into consideration when designing efficacy studies for therapeutics that target the KEAP1/NRF2 axis for MASLD.

## Figures and Tables

**Table 1:** Nucleotide sequence of primers used for analysis byRT-qPCR.

Gene Product	Forward Primer (5′-3′)	Reverse Primer (5′-3′)	Accession Definition
*Nrf2*	GGTTGCCCACATTCCCAAAC	CAGGGCAAGCGACTGAAATG	NM_001399173.1
*Keap1*	GGACGGCAACACTGATTC	TCGTCTCGATCTGGCTCATA	NM_057152.2
*Hmmo1/2*	ACAGGGTGACAGAAGAGGCTAA	CTGTGAGGGACTCTGGTCTTTG	NM_012580
*Nqo1*	AGCCCTGATTGTATTGGCCC	GATTCGACCACCTCCCATCC	NM_017000
*XBP1-s*	GTCCGCAGCACTCAGACTAC	ATCTGAAGAGGCAACAGCGT	NM_001004210.2
*XBP1-u*	CCATGGATTCGGCCCTCAG	CCGAAGAAGATGGGCAGCA	NM_001399536.1
*Hrd1*	CCTGTGAGCACTGCAGAAGA	TGCAAACAGAGAGGGAGCTG	NM_001100739.1
*Beta-actin*	CGCGAGTACAACCTTCTTGC	ATACCCACCATCACACCCTG	NM_031144.3
*Gapdh*	CTTCTTGTGCAGTGCCAGCC	CAAGAGAAGGCAGCCCTGGT	NM_017008.4

## Data Availability

The original contributions presented in the study are included in the article and Supplementary Materials; further inquiries can be directed to the corresponding authors.

## Supplementary Materials

The following supporting information can be downloaded at: https://www.mdpi.com/article/10.3390/cells13151247/s1, Figure S1: Effect of HFHFr diet on the expression levels of unfolded protein response stress sensor XBP1 in the rat livers; Figure S2: The full-size original immunoblots for cytosolic NRF2 IB image; Figure S3: The full-size original immunoblots for nuclear NRF2 IB image; Figure S4: original immunoblots for KEAP1 IB image; Figure S5: The full-size original immunoblots for NQO1 IB image; Figure S6: The full-size original immunoblots for HO1 IB image; Figure S7: The full-size original immunoblots for p62 and LC3B-I and LC3B-II IB images; Figure S8: The full-size original immunoblots for LAMP2_ images; Table S1: Basic characteristics of Sprague Dawley rats after three months of feeding the HFHFr diet.
