# Supplementary material for: Sex Differences Affect the NRF2 Signaling Pathway in the Early Phase of Liver Steatosis: A High-Fat-Diet-Fed Rat Model Supplemented with Liquid Fructose
Source: Cells. 2024 Jul 24;13(15):1247. doi: 10.3390/cells13151247 (PMC11312139; doi:10.3390/cells13151247)
Supplement: Supplementary file 1 [file cells-13-01247-s001.zip › cells-3062121-supplementary.pdf]

Supplementary information

Supplementary Table S1

Table S1. Basic characteristics of Sprague Dawley rats after three months of feeding the HFHFr diet

	**Female rats**		**Male rats**	
	Ctrl	HFHFr	Ctrl	HFHFr
**Final body weight (g)**	260 ± 11	268 ± 9	430 ± 14	438 ± 37
**Liver weight (g)**	7.9 ± 0.6	9.5 ± 1.5 *	15.7 ± 1.5	16.3 ± 1.8
**pWAT weight (g)**	2.2 ± 0.3	2.5 ± 0.5	4.8 ±0.9	7.5 ±2.1*
**Blood TG (mg/dL)**	118 ± 15	197 ± 65 *	109 ± 19	249 ± 57 ***
**Liver TG (mg/g protein)**	50.7 ± 11.4	115.3 ± 60.2**	44.7 ± 8.4	59.6 ± 8.7 **
**Liver Chol (mg/g protein)**	9.9 ± 1.1	11.0 ± 2.1	6.9 ± 1.5	7.3 ± 1.0
**Serum AST (U/L)**	43 ± 6	37 ± 13	40 ± 16	36 ± 8
**Serum ALT (U/L)**	26 ± 6	25 ± 6	29 ± 7	14 ± 6***

Data obtained from control and high-fat high-fructose fed male and female rats (n = 7–8 per group) [27,28]. Data are presented as the mean ± SD. *** $p < 0.001$, ** $p < 0.01$, * $p < 0.05$. ALT: alanine transaminase; AST: aspartate transaminase; Chol: cholesterol; Ctrl: control; pWAT: perigonadal white adipose tissue; TG: triglyceride.

Supplementary information

Supplementary figure S1

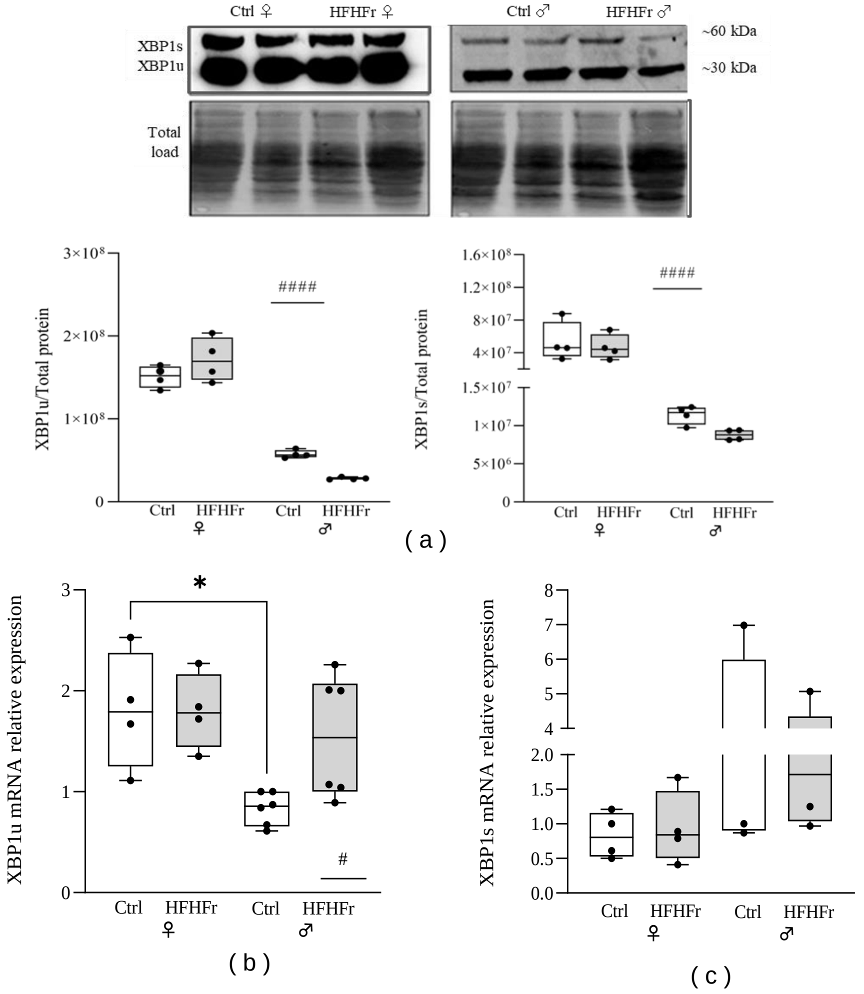

Figure S1. Effect of HFHFr diet on the expression levels of unfolded protein response stress sensor XBP1 in the rat livers. Western blot representative images and densitometric analysis of (a) XBP1u, and XBP1s, and (b) XBP1u and (c) XBP1s mRNA splicing as analyzed by RT-qPCR on isolated mRNA from liver tissue. A significant main effect of sex was observed when both XBP1 and XBP1s were analyzed with male subjects being characterized by higher protein levels than females (XBP1u, sex: $F(1, 12) = 248.7$; $p < 0.0001$; sex x diet interaction: $F(1, 12) = 11.11$; $p = 0.0060$; XBP1s, sex: $F(1, 12) = 31,41$; $p = 0.0001$; sex x diet interaction: $F(1, 12) = 0.06543$; $p = 0.8024$). Sex differences were also observed at the mRNA level (XBP1u, sex: $F(1, 12) = 6.761$; $p = 0.0232$; sex x diet interaction: $F(1, 12) = 2.364$; $p = 0.1501$; XBP1s, sex: $F(1, 12) = 4.230$; $p = 0.0621$; sex x diet interaction: $F(1, 12) = 0.1677$; $p = 0.6894$) with male subjects showing lower levels. Statistical analysis was conducted using two-way ANOVA followed by Tukey's post hoc test for multiple comparisons. Significant differences are indicated by p-values: * $p < 0{,}032$; $^{####}p < 0.0001$

Supplementary information

Supplementary figure S2

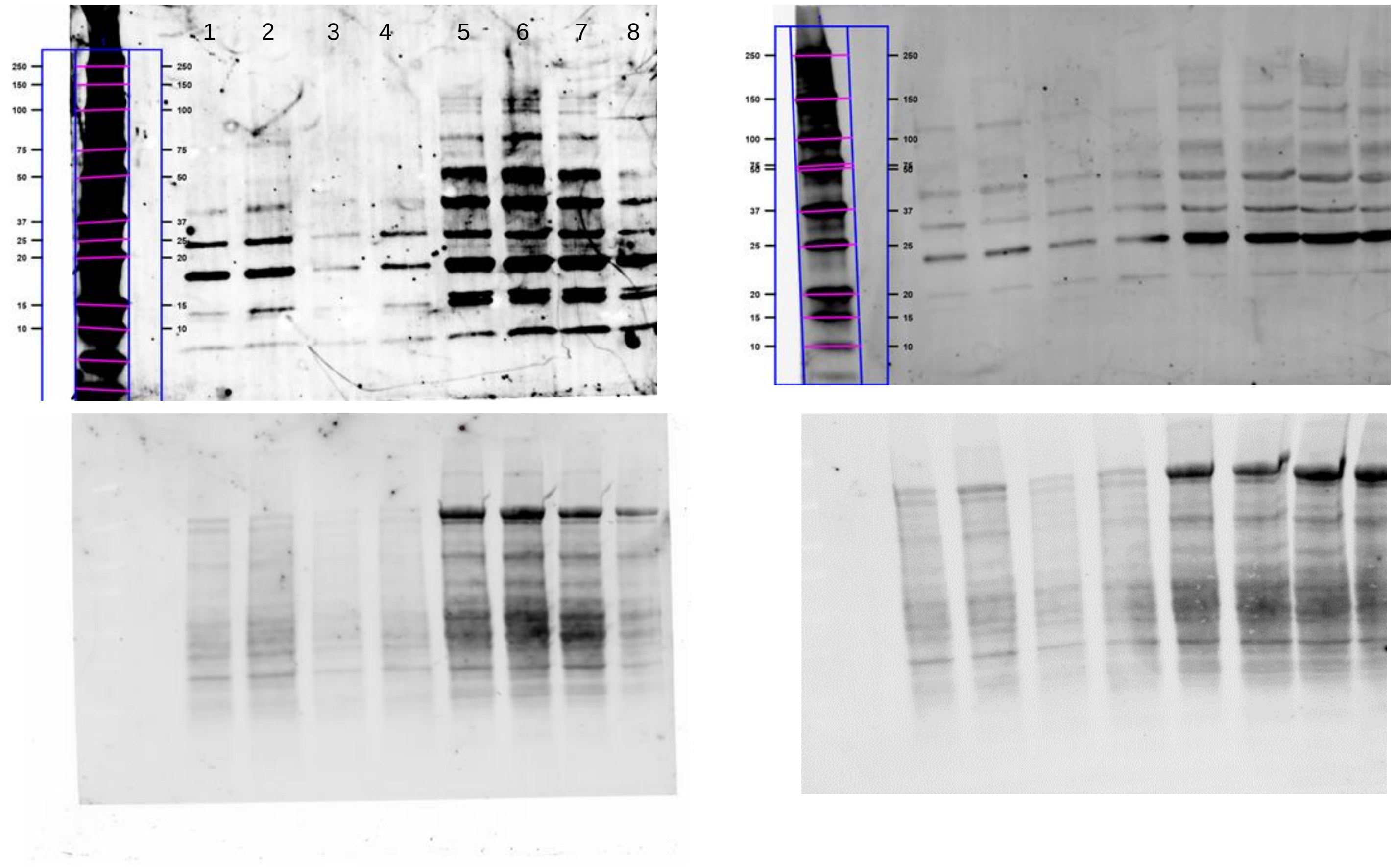

1: Ctrl ♀ pool 1-2
2: Ctrl ♀ pool 3-4
3: Ctrl ♀ pool 5-6
4: Ctrl ♀ pool 7-8
5: HFHFr ♀ pool 1-2
6: HFHFr ♀ pool 3-4
7: HFHFr ♀ pool 5-6
8: HFHFr ♀ pool 7-8

The full-size original immunoblots for cytosolic NRF2 IB image

1: Ctrl ♂ pool 1-2
2: Ctrl ♂ pool 3-4
3: Ctrl ♂ pool 5-6
4: Ctrl ♂ pool 7-8
5: HFHFr♂ pool 1-2
6: HFHFr ♂ pool 3-4
7: HFHFr ♂ pool 5-6
8: HFHFr ♂ pool 7-8

Supplementary information

Supplementary figure S3

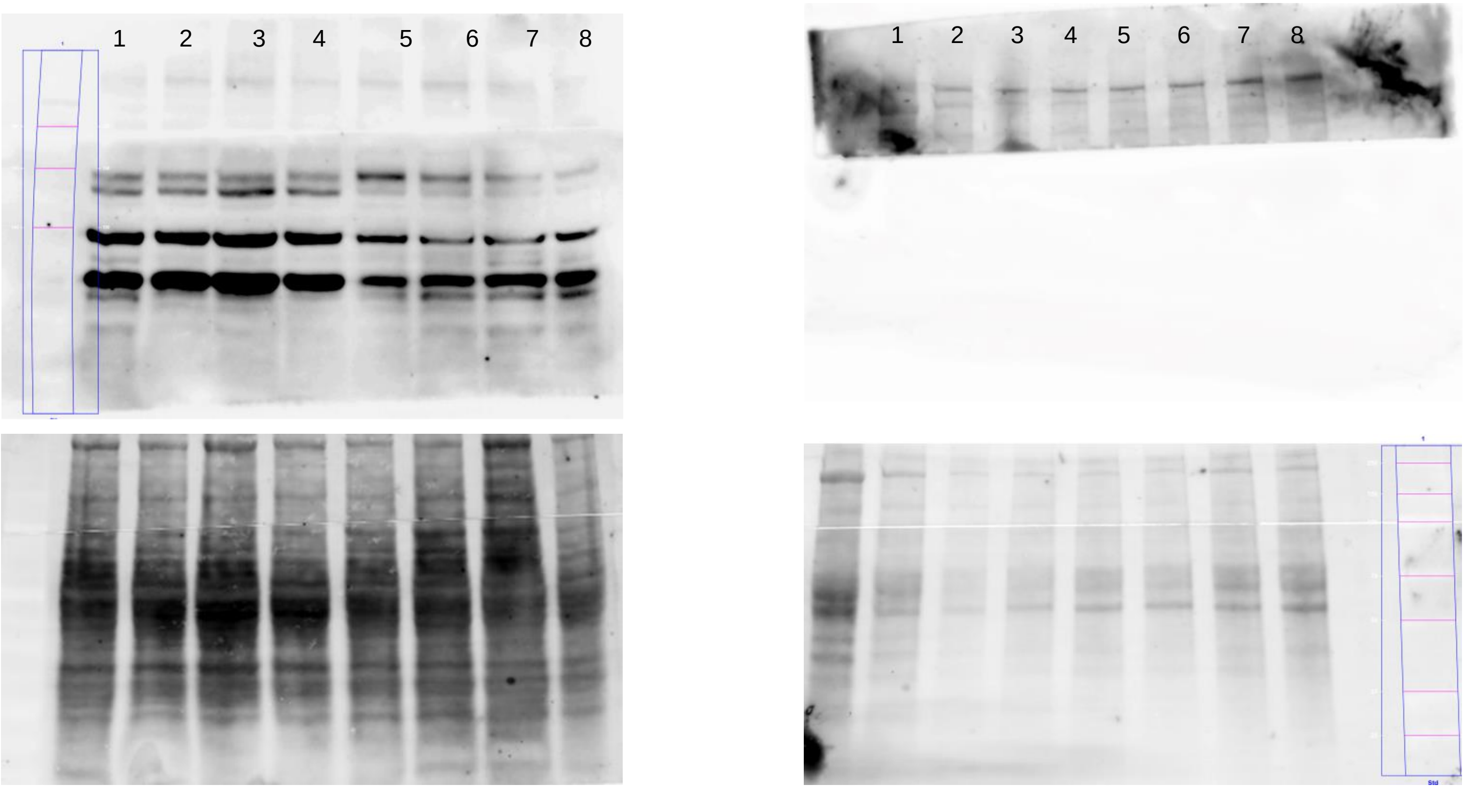

1: Ctrl ♀ pool 1-2
2: Ctrl ♀ pool 3-4
3: Ctrl ♀ pool 5-6
4: Ctrl ♀ pool 7-8
5: HFHFr ♀ pool 1-2
6: HFHFr ♀ pool 3-4
7: HFHFr ♀ pool 5-6
8: HFHFr ♀ pool 7-8

The full-size original immunoblots for nuclear NRF2 IB image

1: Ctrl ♂ pool 1-2
2: Ctrl ♂ pool 3-4
3: Ctrl ♂ pool 5-6
4: Ctrl ♂ pool 7-8
5: HFHFr♂ pool 1-2
6: HFHFr ♂ pool 3-4
7: HFHFr ♂ pool 5-6
8: HFHFr ♂ pool 7-8

Supplementary information

Supplementary figure S4

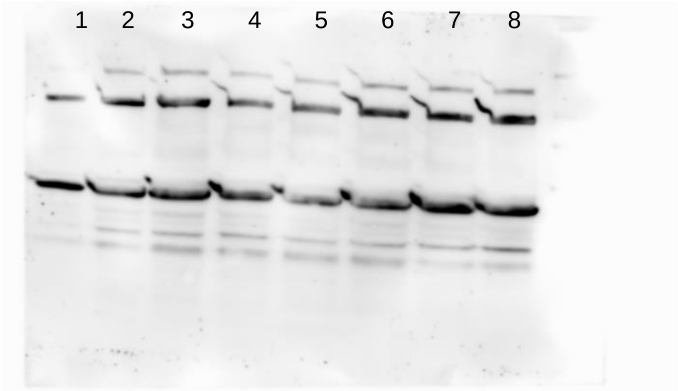

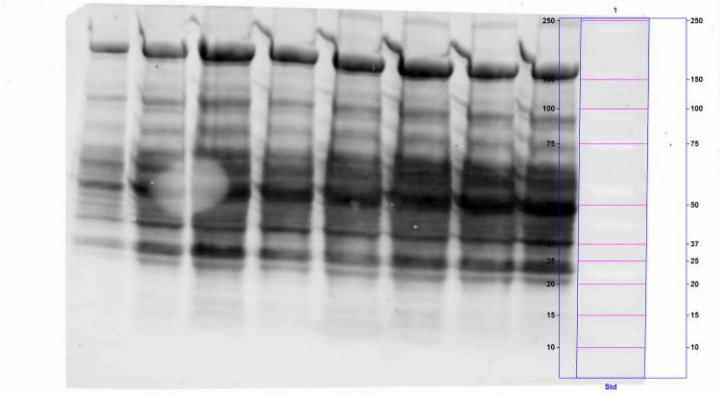

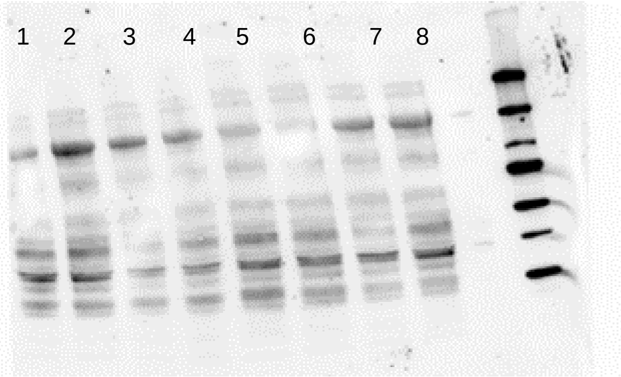

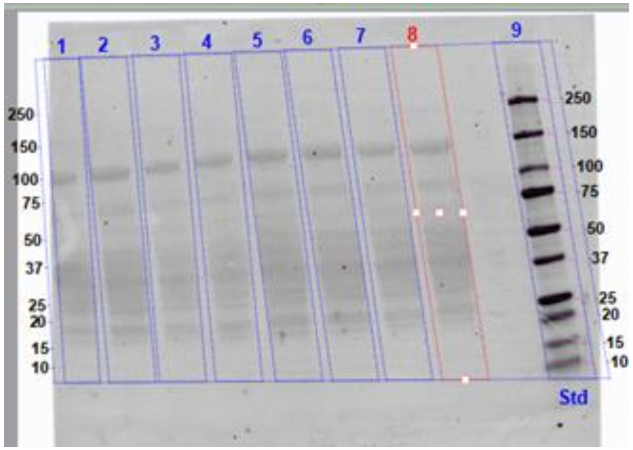

1: Ctrl ♀ pool 1-2
2: Ctrl ♀ pool 3-4
3: Ctrl ♀ pool 5-6
4: Ctrl ♀ pool 7-8
5: HFHFr ♀ pool 1-2
6: HFHFr ♀ pool 3-4
7: HFHFr ♀ pool 5-6
8: HFHFr ♀ pool 7-8

iginal immunoblots for KEAP1 IB image

1: CT ♂ pool 1-2
2: CT ♂ pool 3-4
3: CT ♂ pool 5-6
4: CT ♂ pool 7-8
5: HFHFr ♂ pool 1-2
6: HFHFr ♂ pool 3-4
7: HFHFr ♂ pool 5-6
8: HFHFr ♂ pool 7-8

Supplementary information

Supplementary figure S5

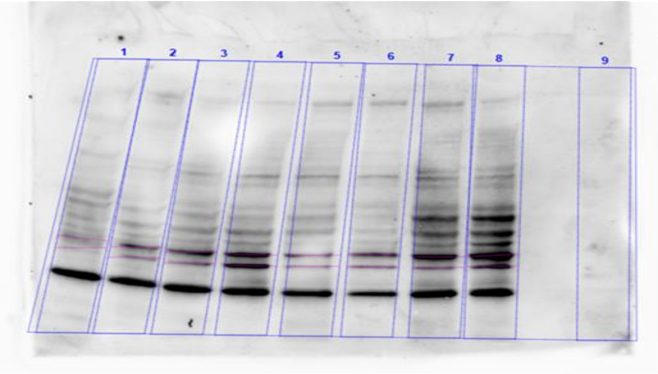

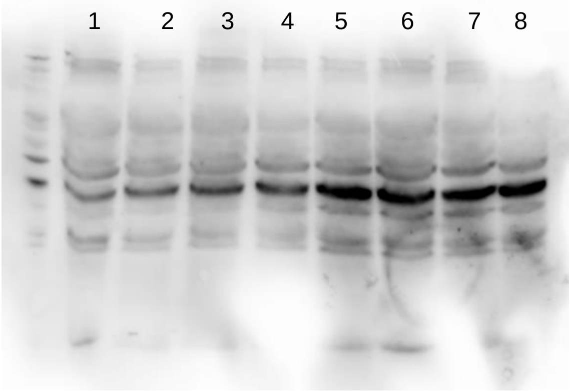

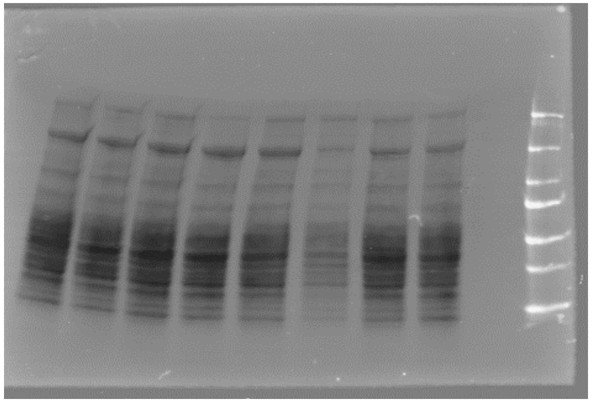

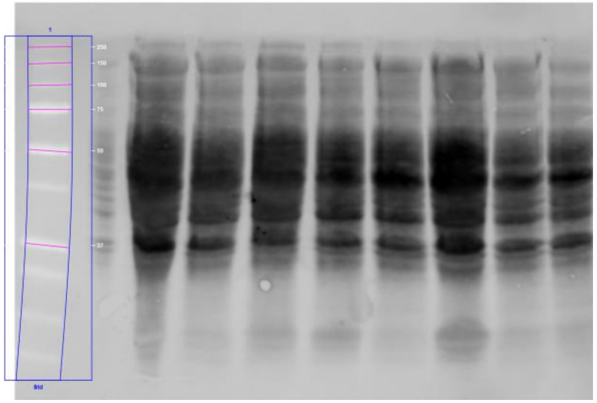

1: Ctrl ♀ pool 1-2
2: Ctrl ♀ pool 3-4
3: Ctrl ♀ pool 5-6
4: Ctrl ♀ pool 7-8
5: HFHFr ♀ pool 1-2
6: HFHFr ♀ pool 3-4
7: HFHFr ♀ pool 5-6
8: HFHFr ♀ pool 7-8

The full-size original immunoblots for NQO1 IB image

1: HFHFr ♂ pool 1-2
2: HFHFr ♂ pool 3-4
3: HFHFr ♂ pool 5-6
4: HFHFr ♂ pool 7-8
5: CT ♂ pool 1-2
6: CT ♂ pool 3-4
7: CT ♂ pool 5-6
8: CT ♂ pool 7-8

Supplementary information

Supplementary figure S6

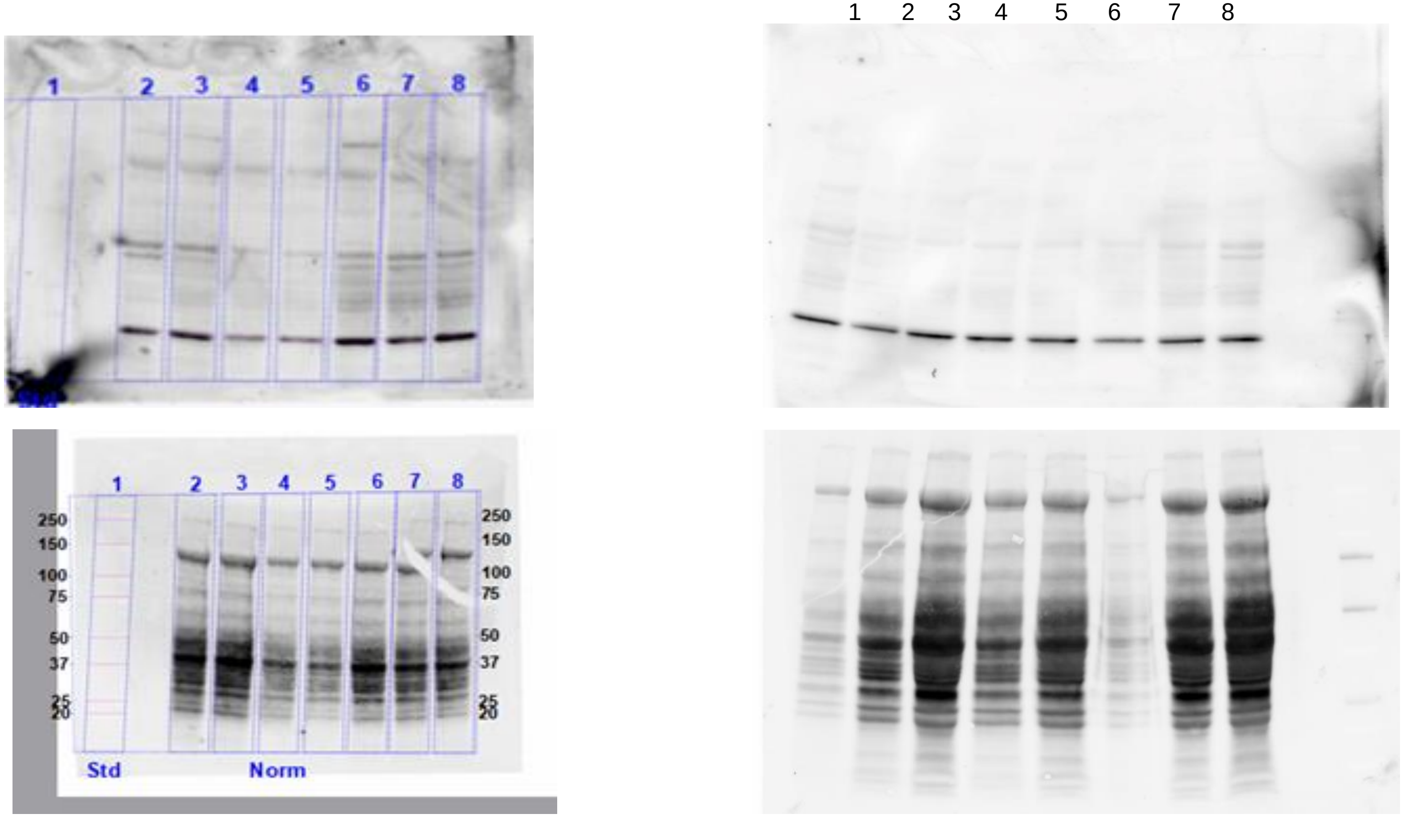

1: Ctrl ♀ pool 1-2
2: Ctrl ♀ pool 3-4
3: Ctrl ♀ pool 5-6
4: Ctrl ♀ pool 7-8
5: HFHFr ♀ pool 1-2
6: HFHFr ♀ pool 3-4
7: HFHFr ♀ pool 5-6
8: HFHFr ♀ pool 7-8

The full-size original immunoblots for HO1 IB image

1: CT ♂ pool 1-2
2: CT ♂ pool 3-4
3: CT ♂ pool 5-6
4: CT ♂ pool 7-8
5: HFHFr ♂ pool 1-2
6: HFHFr ♂ pool 3-4
7: HFHFr ♂ pool 5-6
8: HFHFr ♂ pool 7-8

Supplementary information

Supplementary figure S7

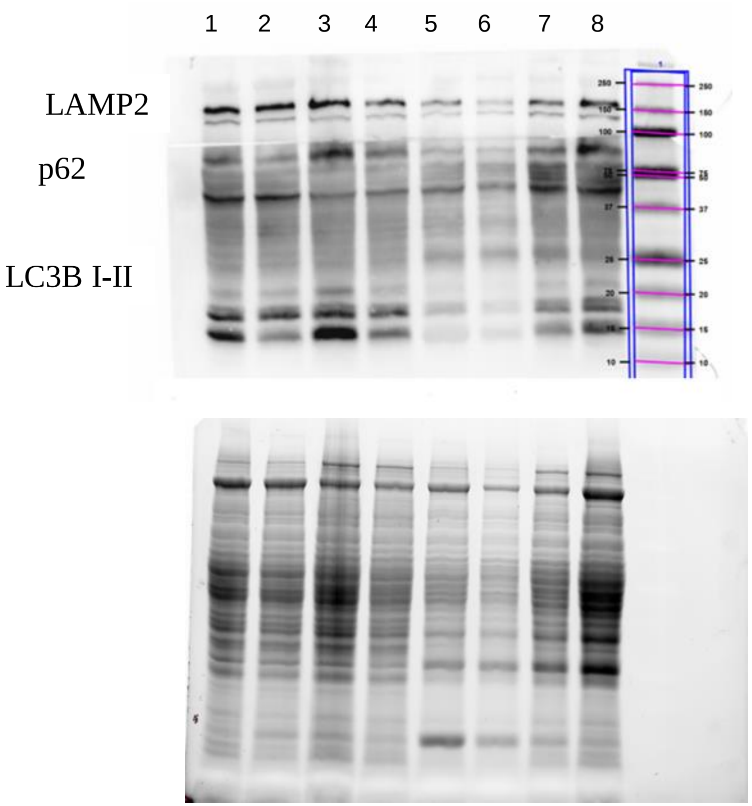

1: Ctrl ♀ pool 1-2
2: Ctrl ♀ pool 3-4
3: HFHFr ♀ pool 1-2
4: HFHFr ♀ pool 3-4
5: Ctrl ♂ pool 1-2
6: Ctrl ♂ pool 3-4
7: HFHFr ♂ pool 1-2
8: HFHFr ♂ pool 3-4

The full-size original immunoblots for p62 and LC3B-I and LC3B-II IB images

Supplementary information

Supplementary figure S8

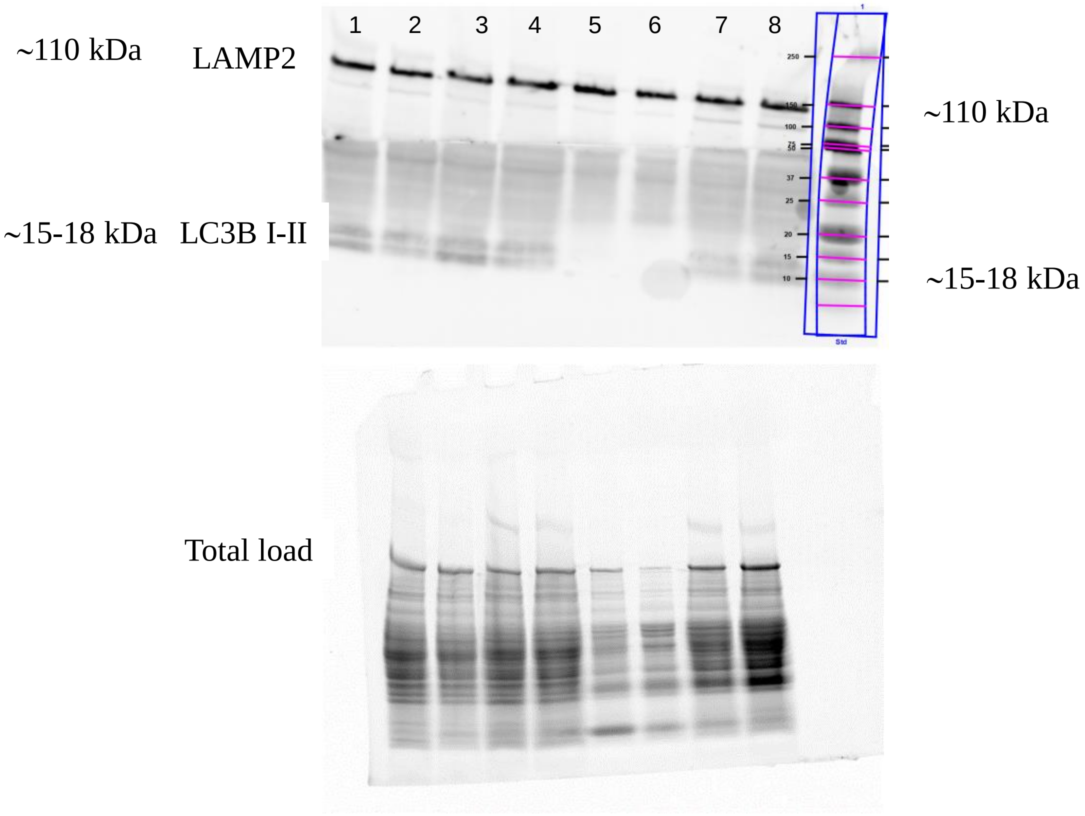

1: Ctrl ♀ pool 1-2
2: Ctrl ♀ pool 3-4
3: HFHFr ♀ pool 1-2
4: HFHFr ♀ pool 3-4
5: Ctrl ♂ pool 1-2
6: Ctrl ♂ pool 3-4
7: HFHFr ♂ pool 1-2
8: HFHFr ♂ pool 3-4

The full-size original immunoblots for LAMP2_ images
